# Normalization and Technical Variation in Gene Expression Measurements

**DOI:** 10.6028/jres.111.026

**Published:** 2006-10-01

**Authors:** Walter Liggett

**Affiliations:** National Institute of Standards and Technology, Gaithersburg, MD 20899-8980

**Keywords:** calibration linearity, classifier performance, high-density microarrays, inter-laboratory study, latent variables, MAQC, microarray quality control, simultaneous F tests, sources of variation, statistical dependence

## Abstract

Using data from the Microarray Quality Control (MAQC) project, we demonstrate two data-analysis methods that shed light on the normalization of gene expression measurements and thereby on their technical variation. One is an improved method for normalization of multiple assays with mRNA concentrations related by a parametric model. The other is a method for characterizing limitations on the effectiveness of normalization in reducing technical variation. We apply our improved normalization to the four project materials as part of testing the linearity of the probe responses. We find that the lack of linearity is statistically significant but small enough that its sources cannot be easily identified. Applying our characterization method to assays of the same material, we show that there is a source of variation that cannot be eliminated by normalization and therefore must be dealt with by other means. Four high-density, single probe, one-color microarray platforms underlie our demonstration.

## 1. Introduction

Monitoring the expression of thousands of genes simultaneously for the purpose of obtaining biological insight requires an effective approach to the technical variation. Particulars of technical variation are often sought through an inter-laboratory study, of which the Microarray Quality Control (MAQC) project is an example. Using data from the MAQC project, we demonstrate statistical modeling techniques that lead to better understanding of the technical variation in gene expression assays. These techniques have had little previous application in microarray data analysis. In Sec. 4, we discuss the relation of conclusions based on these techniques to other MAQC results.

In the MAQC project, gene expression levels were measured from two high-quality, distinct RNA reference materials and two mixtures of these materials. Data from four microarray platforms of the seven in the MAQC project provide the basis for the demonstrations in this paper. These platforms are^1^

**Table:** 

Manufacturer	Code	Platform
Applied Biosystems	ABI	Human Genome Survey Microarray v2.0
Illumina	ILM	Human-6 BeadChip 48K v1.0
Agilent	AG1	Whole Human Genome Oligo Microarray, G4112A
GE Healthcare	GEH	CodeLink Human Whole Genome, 300026

With some extensions, our techniques could be applied to the data from the other three microarray platforms. Each microarray platform was deployed at three test sites, and five replicates were assayed at each site. Thus, for each platform, the MAQC project produced sixty assays. The complicating feature in this otherwise simple inter-laboratory study is the large number of expression levels measured in each assay. This feature leads us to the use of novel statistical methods for investigating technical variation.

In its original form, a gene expression assay consists of many probe responses, which are generally intensity readings from a scanner. In the cases of the platforms considered here, the number of probe responses is greater than 30,000, although we only select some of these responses for our demonstrations. Each probe is a distinct piece of nucleic acid that is immobilized on the microarray substrate. In the case of gene expression, each probe interrogates an mRNA sample to determine the relative abundance of a particular transcript among all the transcripts that make up the sample. Hybridization is the process of applying an mRNA sample to the probes that make up a microarray.

Sources of technical variation, the focus of this paper, affect the probe responses. Some sources of technical variation affect most probe responses shifting all of them up or all of them down. The effects of such sources can be corrected in part through what is called normalization. Two basic choices of normalization method are usually considered. One is assay-to-assay equalization of the intensity distribution [1]. The other is use of external RNA controls [2, 3]. The part of the technical variation that is most important is the part that normalization cannot correct.

A third normalization choice can be applied to probe responses from the MAQC project. Each laboratory measured four reference materials, two of which are mixtures of the other two. Because of this mixing, the expression levels in the materials are all related by a parametric model: The expression levels in the mixture materials are known proportions of the levels in the original materials. As this paper demonstrates, this parametric model can be the basis for a different type of normalization. Although this normalization is potentially useful in reaching biological conclusions, our immediate purpose for introducing it is testing the linearity of the probe responses.

Another aspect of normalization is limitations on its effectiveness. The basis of normalization, regardless of the type, is assumption of a relation among the effects of sources of technical variation on the intensity measurements from an array. Equalization of intensity distributions depends, at a minimum, on the assumption that the technical variation shifts all the intensities of an assay in the same direction. Use of external controls depends on an assumption that allows changes in the control intensities to be extrapolated to the other intensities. In this paper, we use the MAQC measurements to show limitations on normalization due to more complicated intensity co-variation.

This paper provides results on the technical variation observed through measurements of different materials made in the same laboratory and through measurements of the same material made in three different laboratories. The two sets of results are based on different statistical models, which are discussed in the next section. The results themselves appear in Sec. 3 and are discussed in Sec. 4. Finally, details on the methods are given in Sec. 5.

## 2. Statistical Models

Implementation of our normalization method, which we call parametric normalization, requires a mathematical model that faithfully describes the relations among assays. For a particular laboratory, consider the intensity value (probe response) for assay *i* and probe *g*, which we denote by *y_ig_*, where *i* = 1, …, 20. The 20 assays are 5 hybridizations of material A, 5 of material B, 5 of material C, and 5 of material D, respectively. Parametric normalization as implemented here consists of a linear transformation of all the intensities of an assay. Thus, after normalization, the intensity for assay *i* and probe *g* is given by

$$\frac{y_{ig}-\eta_{0i}}{\eta_{i}},$$

where *η*_0_*_i_* and *η_i_* are the normalization coefficients for assay *i*. Our parametric normalization model is

$$(y_{ig}-\eta_{0i})/\eta_{i}=x_{Ai}\theta_{Ag}+x_{Bi}\theta_{Bg}+\sigma_{ig}e_{ig},$$ (1)

where for materials A, B, C and D, *x_Ai_* equals 1, 0, *γ_C_* or (1 – *γ_D_*), respectively, and *x_Bi_* equals 0, 1, (1 – *γ_C_*) or *γ_D_*, respectively. This equation represents intensities in terms of mean intensities, *θ_Ag_* and *θ_Bg_*, and noise. For material A, the right side is *θ_Ag_* + *σ_ig_e_ig_*, and for material B, *θ_Bg_* + *σ_ig_e_ig_*. The quantity *e_ig_* is a random variable with mean zero and variance one. The standard deviation of the noise *σ_ig_* is shown as a function of assay *i* and probe *g*. In this model, the fraction of material C that came from material A is *γ_C_*, and the fraction of material D that came from material B is *γ_D_*. On the basis of this equation, parametric normalization is performed by fitting the unknown parameters *η*_0_*_i_*, *η_i_*, *θ_Ag_*, and *θ_Bg_* to the observed intensities *y_ig_* and then using the estimated values of *η*_0_*_i_* and *η_i_* for normalization. The algorithm is given in Sec. 5.

Investigation of the limitations on normalization also requires a mathematical model but of a different type. Consider measurements of the same material given as intensities on the base 2 logarithmic scale. We denote these intensities by *z_ig_*, where *i* = 1, …, 15 indexes the five assays from each of three laboratories. Computation of the correlation matrix is a familiar summarization of such data. Let the covariance between assays *i* and *j* be

$$s_{ij}=\frac{1}{G-1}\sum_{g}\left(z_{ig}-{\bar{z}}_{i})(z_{jg}-{\bar{z}}_{j}\right),$$ (2)

where ${\bar{z}}_{i}$ is the mean of the log intensities over the probes for assay *i* and *G* is the number of probes. The familiar correlation between assays *i* and *j* is given by

$$s_{ij}/\sqrt{s_{ii}s_{jj}}.$$

Our approach is based on modeling the covariance between assays *i* and *j*, which is estimated by *s_ij_*, using a factor analysis model with two common factors. The covariance model is

$$\begin{array}{ll} \lambda_{i1}^{2}+\lambda_{i2}^{2}+\psi_{i} & \text{if}\;i=j\\ \lambda_{i1}\lambda_{j1}+\lambda_{i2}\lambda_{j2} & \text{if}\;i\neq j. \end{array}$$

This covariance modeling is equivalent to modeling the *z_ig_* by means of the factor analysis model

$$z_{ig}=\mu_{i}+\lambda_{i1}f_{1g}+\lambda_{i2}f_{2g}+\psi_{i}^{1/2}e_{ig},$$ (3)

where the *f*_1_*_g_*, *f*_2_*_g_*, and *e_ig_*, *i* = 1, …, 15 are all independent, normal random variables with mean 0 and variance 1. In this model, the random variables *f*_1_*_g_* and *f*_2_*_g_*, represent the probe-to-probe variation in the common factors and the parameters *λ_i_*_1_ and *λ_i_*_2_ govern the contributions of the common factors to the individual assays. This modeling gives the (log) intensity distribution for assay *i* as normal with mean *µ_i_* and variance $\lambda_{i1}^{2}+\lambda_{i2}^{2}+\psi_{i}$. The limitation arises because equalization of the intensity distribution has only this mean and variance available for adjustment and not the *λ_i_*_1_ and *λ_i_*_2_ individually.

The results in this paper are based on modeling the technical variation in four platforms. From platform to platform, the technical variation as portrayed by these two models is similar. We leave aside gene-level modeling of the differences among platforms [4]. We do not consider the question of inter-platform reproducibility, the main subject of many studies [5, 6].

## 3. Results

### 3.1 Parametric Normalization

To assess the linearity of the probe responses after parametric normalization, we determine how well the mixture model fits the normalized measurements. Our measure of lack of fit is a weighted sum of squared deviations, which we compute for each probe. For a particular laboratory, let the normalized intensities for probe *g* be denoted by *u_ig_*, *i* = 1, …, 20. From these intensities, we estimate *θ_Ag_* and *θ_Bg_* denoting the estimates by ${\hat{\theta }}_{Ag}$ and ${\hat{\theta }}_{Bg}$. The averages of the five normalized intensities for each material deviate from the values computed with the mixture model in accordance with

$$\begin{array}{c} {\bar{u}}_{Ag}-{\hat{\theta }}_{Ag}\\ {\bar{u}}_{Bg}-{\hat{\theta }}_{Bg}\\ {\bar{u}}_{Cg}-\gamma_{C}{\hat{\theta }}_{Ag}-(1-\gamma_{C}){\hat{\theta }}_{Bg}\\ {\bar{u}}_{Dg}-(1-\gamma_{D}){\hat{\theta }}_{Ag}-\gamma_{D}{\hat{\theta }}_{Bg} \end{array}.$$

Note that these deviations reflect departure of the normalized probe responses from linearity. Lack-of-fit for each probe is indicated by the weighted sum of squares of these deviations

$$\begin{array}{l} w_{Ag}{\left({\bar{u}}_{Ag}-{\hat{\theta }}_{Ag}\right)}^{2}+w_{Bg}{\left({\bar{u}}_{Bg}-{\hat{\theta }}_{Bg}\right)}^{2}\\ +w_{Cg}{\left({\bar{u}}_{Cg}-\gamma_{C}{\hat{\theta }}_{Ag}-(1-\gamma_{C}){\hat{\theta }}_{Bg}\right)}^{2}\\ +w_{Dg}{\left({\bar{u}}_{Dg}-(1-\gamma_{D}){\hat{\theta }}_{Ag}-\gamma_{D}{\hat{\theta }}_{Bg}\right)}^{2}, \end{array}$$

where the weights *w_Ag_*, *w_Bg_*, *w_Cg_*, and *w_Dg_* are inversely proportional to *σ_ig_*^2^.

The amounts of RNA material mixed to form materials C and D were in 3 to 1 and 1 to 3 ratios. Were the fraction of mRNA in materials A and B the same, we would have *γ_C_* = *γ_D_* = 0.75. However, these fractions are not exactly equal. Based on our analysis of the MAQC data and a complementary analysis [7], we have adopted *γ_C_* = 0.80 and *γ_D_* = 0.69 as values for our deviations. In our analysis, we obtained estimates of *γ_C_* and *γ_D_* from each of the four platforms we consider. The estimates given by different platforms are in reasonable agreement.

One way to interpret our deviations is to compare them with what would be expected were the mixture model to fit perfectly [8]. We obtain a standard deviation from each of the five normalized intensities for a material and denote these by *s_Ag_*, *s_Bg_*, *s_Cg_*, and *s_Dg_*. The comparison involves the ratio of the sum of squared deviations with

$$\frac{1}{10}\left[w_{Ag}s_{Ag}^{2}+w_{Bg}s_{Bg}^{2}+w_{Cg}s_{Cg}^{2}+w_{Dg}s_{Dg}^{2}\right].$$

The scale factor 1/10 depends on specifics of the MAQC design and the number of unknown parameters in the mixture model. If the mixture model fits, this ratio is F distributed, that is, distributed as the ratio of two independent variances. The numerator and denominator degrees of freedom are 2 and 16.

**Fig. 1:**
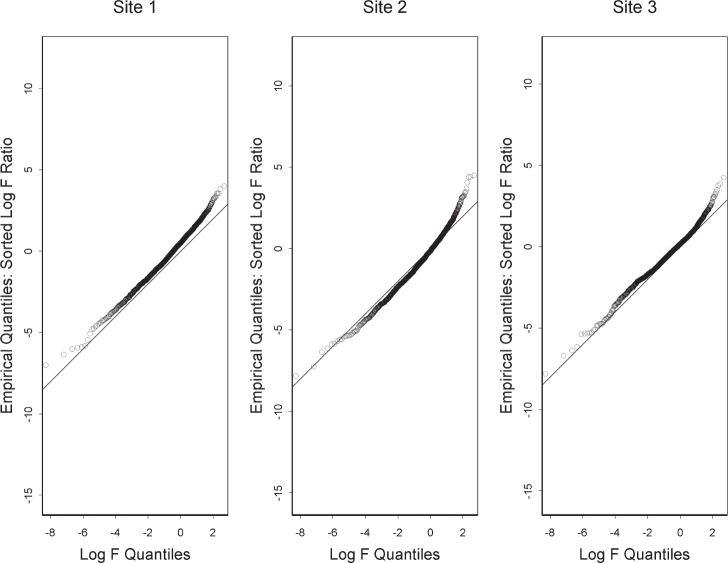
For ABI, quantile-quantile plots that compare the observed F statistics with the F distribution with 2 and 16 degrees of freedom. Closeness to the *x* = *y* line indicates weakness of evidence of lack of fit.

To complete this interpretation of lack-of-fit, we combine the F values from all the probes into a distribution and compare this observed distribution with the F distribution for 2 and 16 degrees of freedom. We make this comparison with a quantile-quantile plot. Each point on this plot corresponds to a probe. The y value is the natural logarithm of the F value for this probe. Thus, a *y* value corresponds to an observed quantile. The corresponding *x* value is obtained from the ranking of this F value among all the F values. The *x* value is obtained as follows: First, find the proportion of all the F values that are less than the particular F value. Let this proportion be *m/n*, where *n* is the total number of probes. Second, find the value such that the cumulative F distribution for this value is (*m* + 0.5)/*n*. The logarithm of this value is the *x* value for the probe. Thus, an *x* value corresponds to a quantile hypothesized on the basis of perfect fit to the F distribution. Quantile-quantile plots for the sites that made use of the Applied Biosystems platform (ABI) are shown in Fig. 1. The methods section describes selection of probes for this figure. In addition to the points, there is the *x* = *y* line. If there were no lack of fit, the points would lie on this line. Apparently, there is some lack of fit, and the unanswered questions involve the implications of this lack of fit.

The size of the gap between the points and the *x* = *y* line depends on both the fit of the mixture model and on the denominator of the F ratio, which is proportional to the variance exhibited by the replicate measurements on the same material. To judge the gap in terms of the replicate variance, we can ask for the size of the factor that the denominator of the F ratio would have to increase for the points and the line to coincide. This factor is generally small as is shown by the fact that if the denominator were increased by a factor of 2.7, then the points would move downward 1 unit on the *y* axis. This suggests that the lack of fit exhibited in Fig. 1 is so small in comparison to the noise level that diagnosis of this lack of fit might be difficult. Moreover, note that the gap is ambiguous as a performance measure since it increases with lack of fit but decreases with increasing noise level.

For each of the sites in Fig. 1, the departure of the points from the *x* = *y* line is greater at the upper end. This shows that the upper tail of the observed distribution is heavier than it would be were the F values truly F distributed. Generally, one might summarize the lack of fit as being most notable in a small fraction of the probes that have observed F values so large that they cannot be attributed to estimation error as given by the F distribution.

Another view of the sum of squared deviations is comparison of its value under parametric normalization with its value under the manufacturer’s specified normalization. To obtain this latter value, we use the manufacturer’s values for *u_ig_* but do not change the weighting in either the estimation of *θ_Ag_*, and *θ_Bg_* and or in the computation of the weighted sum of squared deviations. Because the two normalizations may differ by a scale factor, we divide each sum of squares by the corresponding value of ${\left({\hat{\theta }}_{Ag}-{\hat{\theta }}_{Bg}\right)}^{2}$. We have compared one scaled sum of squares with the other for each of the sites that used the Illumina platform (ILM). We see that for the majority of the probes considered, parametric normalization gives a smaller scaled sum of squares, which indicates that parametric normalization provides a better fit. This, of course, is not surprising because the parametric normalization is optimized on the basis of the mixture model whereas the manufacturer’s normalization does not make use of such knowledge.

**Fig. 2:**
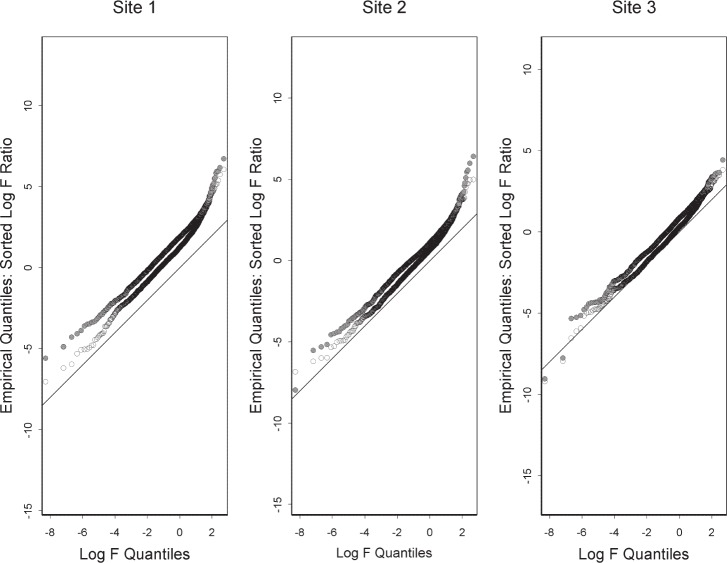
For ILM data under parametric normalization (black open circle) and under manufacturer’s prescribed normalization (gray solid circle), superimposed quantile-quantile plots that compare the two sets of observed F statistics with the F distribution with 2 and 16 degrees of freedom. Closeness to the *x* = *y* line indicates better linearity for parametric normalization.

**Fig. 3:**
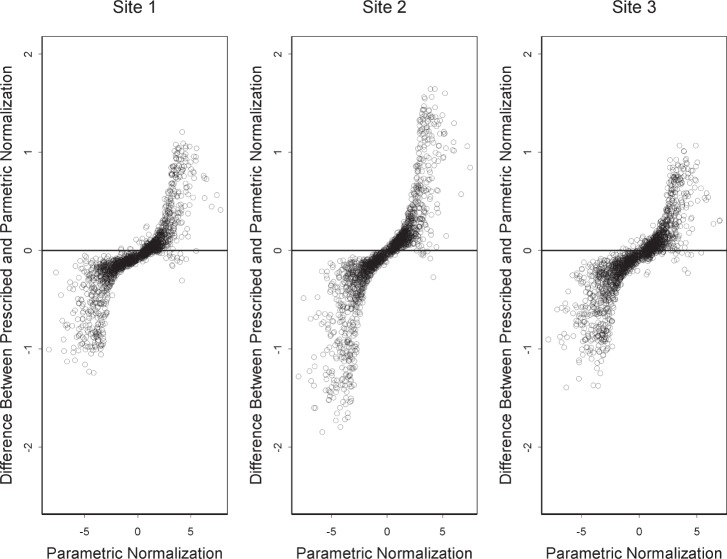
For ILM data, bias in differential expression under manufacturer’s prescribed normalization when compared to parametric normalization. The differences in differential expression values are plotted versus the parametric normalization values.

In characterizing the non-linearity of probe responses, one must apply normalization but, ideally, normalization that does not introduce non-linearity by itself. Figure 2 provides lack-of-fit quantile-quantile plots for the data under parametric normalization and under manufacturer’s prescribed normalization. That the parametric normalization gives F statistics closer to the *x* = *y* line shows that parametric normalization is better for characterization of the inherent non-linearity of the probe responses. Linearity of the probe responses is especially important in the determination of differential expression between two samples. We consider the differential expression between materials A and B expressed as the base 2 logarithm of the ratio of the two responses. We compute the differential expression as log_2_ $\left({\hat{\theta }}_{Ag}/{\hat{\theta }}_{Bg}\right)$. Figure 3 compares differential expression determined under parametric normalization with differential expression determined under manufacturer’s prescribed normalization. In Fig. 3, we treat parametric normalization as the baseline because it provides better linearity as shown in Fig. 2. The vertical dimension shows the bias introduced by the manufacturer’s prescribed normalization. Note that the bias is toward higher differential expression for material A. This figure shows that were it possible, there might be benefit in using parametric normalization in the interpretation of the results from a biological experiment.

### 3.2 Effectiveness of Normalization

We now switch from within-laboratory modeling of different materials to among-laboratory modeling of the same material. It has long been recognized that there are potentially substantial inter-laboratory differences in outright measurement of intensities. For this reason, one would avoid comparison of measured intensities across laboratories if one could. However, biological considerations in the design of a study may require processing samples to be compared in different laboratories or in a single laboratory with sufficient time delay that the technical variation will have inter-laboratory characteristics. This sub-section shows the perils in such comparisons.

Of course, the meaningfulness of the following characterization of inter-laboratory differences depends on data used for the characterization. We note the following: Mixing should not be apparent as a source of inter-laboratory variation because the materials were mixed in one laboratory and shipped to all the other sites in the project. Each platform manufacturer was motivated to choose the other two sites for their platform carefully and to harmonize protocols among the three sites. There was a pilot study that contributed to the harmonization. However, it is true that gauging how well the protocols agreed is difficult because of the multidimensional nature of microarray assays. Finally, generalization from three sites per platform is subject to appreciable uncertainty. Thus, from the MAQC data, one cannot say how difficult it might be to overcome the inter-laboratory differences discussed.

As discussed in the methods section, we select, for the Agilent platform (AG1), the 3000 probes for each material that have the highest intensity overall. Applying factor analysis to the set of material A intensities transformed to the log base 2 scale, we obtain Table 1. The symbols heading the columns in Table 1 are those found in Eq. (3). The column labeled Center gives the mean of the 3000 log intensities for each assay. Inclusion of this parameter in the model provides an elementary form of assay-to-assay normalization. The column labeled Specific Factor gives an estimate of the standard deviation of the noise that is independent from probe to probe. This column also shows some difference among laboratories.

The columns labeled Common Factor complete the model of the distribution of the log intensities that our analysis provides. The intensities are centered at *µ_i_* and have spread given by the standard deviation $\sqrt{\lambda_{i1}^{2}+\lambda_{i2}^{2}+\psi_{i}}$. Between assays *i* and *j*, the log intensities have correlation given by

$$\frac{\lambda_{i1}\lambda_{j1}+\lambda_{i2}\lambda_{j2}}{\sqrt{\left(\lambda_{i1}^{2}+\lambda_{i2}^{2}+\psi_{i}\right)\left(\lambda_{j1}^{2}+\lambda_{j2}^{2}+\psi_{j}\right)}}.$$

The amount of correlation depends on the similarity of parameters *λ_i_*_1_ and *λ_i_*_2_, which model how the common sources affect each assay.

**Fig. 4:**
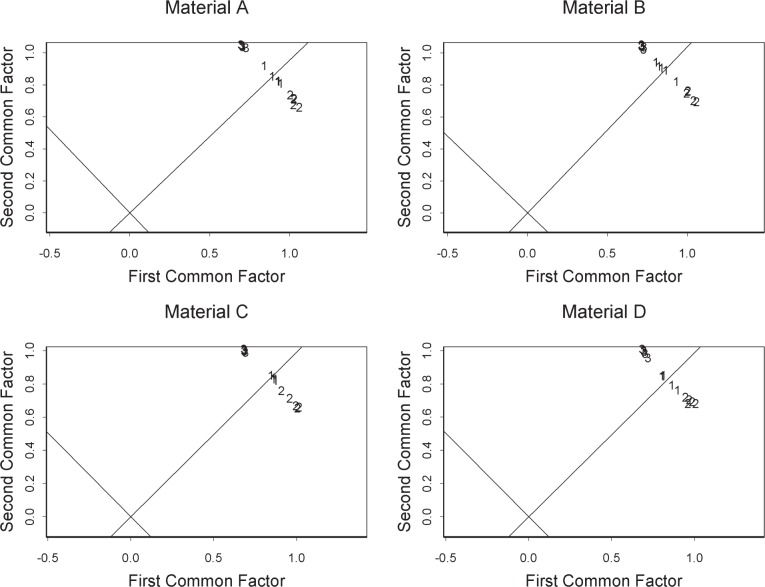
For AG1, plots of common factors showing assay differences with assay sites labeled. Shown are the relations of the differences to the origin. Distribution-based normalization might bring all the assays to the same distance from the origin, but it cannot bring the assays together in the other dimension.

The relation between the components of the two common factors *λ_i_*_1_ and *λ_i_*_2_ is shown in Fig. 4 for all four materials. Normalization is generally based on differences among distributions of the intensities of the assays. In terms of our model, this distribution depends on the distance of the points in this figure from the origin. Thus, normalization can be used to adjust the intensities for each assay so that the distance of each assay from the origin is the same. However, Fig. 4 shows that such adjustment cannot eliminate the differences among assays. The oblique axes shown in each panel generally differentiate the part of the assay-to-assay differences that can and cannot be reduced by normalization. Apparently, there is a source of technical variation that must be dealt with not by normalization, but by other means. Such a source might be variation in some experimental circumstance, perhaps temperature or washing technique, that interacts with the strength of the hybridization bond.

In addition to showing the effects of sources of variation, Fig. 4 also shows clustering of the assays by laboratory, which portrays the magnitude of the among-laboratory variation compared to the within-laboratory variation. Comparisons like this are central to metrology. In the case of univariate measurements, the comparison is between the within-laboratory variance and the among-laboratory variance and is called gauge R&R (repeatability and reproducibility) [9]. In the case of high dimensional measurements such as gene expression assays with thousands of probes, it is common to apply some form of dimension reduction such as principal components analysis (PCA) followed by display of the measurements in the reduced coordinates [10, 11]. Irizarry et al. [12] discuss inter-laboratory variation in gene expression measurements.

Instead of PCA plots, we obtain a perspective on the measurements that places more emphasis on the among-laboratory variation. Our approach is linear discriminant analysis. Like PCA, we find two linear combinations of the measurements from each assay. In the case of discriminant analysis, these combinations are called discriminant coordinates. The difference is that we find linear combinations that maximize the separation of the laboratories with respect to the within-laboratory variation. To do this, we must adopt penalized discriminant analysis [13, 14].

**Fig. 5:**
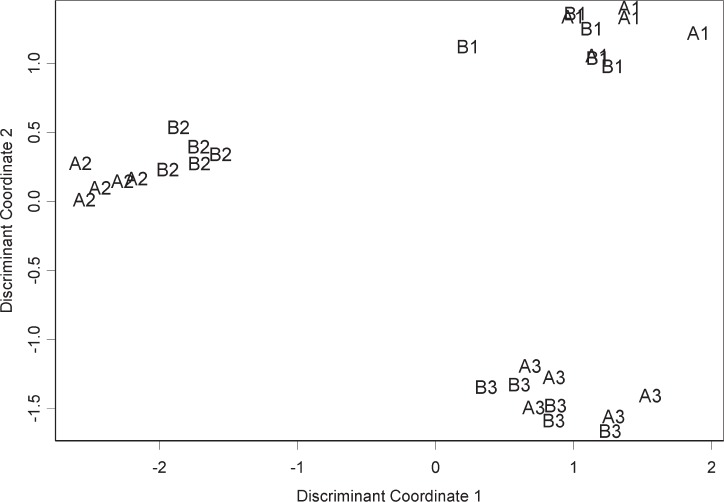
For GEH, assays labeled by material and site plotted with material A discriminate coordinates. Coordinates give the maximum inter-laboratory separation for results on material A. Plot shows that material B results have laboratory signatures like those of material A.

Based on the log_2_ intensities for material A from the GE Healthcare platform (GEH), we derive discriminant coordinates with which we plot the 15 material-A assays. These points along with points for material B are shown in Fig. 5. We see that the groups of material-A points for each laboratory are separated. This is not surprising since we derived the discriminant coordinates from the material A measurements.

What is more interesting about Fig. 5 is the clustering of the material-B points. The discriminant coordinates for material A also separate the material B assays by laboratory. Apparently, the measurements from each array have inherent in them a signature associated with the processing laboratory. Such a signature originates from one or more sources of technical variation. This signature is strong enough that it stands out despite the difference between materials A and B. Were we to add materials C and D to Fig. 5 in the same way as material B, we would obtain the same clustering for these materials. Reversing the roles of materials A and B, that is, deriving the discriminant coordinates from the material B measurements instead, gives a result similar to Fig. 5.

Portraying among-laboratory differences in terms of discriminant analysis is particularly relevant to development of biomarkers on the basis of microarray measurements. Biomarkers can be developed from microarray measurements of samples from both cases and controls. Consider the possibility that the cases have been measured in a different laboratory than the controls, perhaps for logistical reasons. What Fig. 5 shows is that among-laboratory differences could easily be mistaken for case-control differences. Moreover, this suggests that within-laboratory differences involving a considerable time period might also be mistaken for case-control differences.

## 4. Discussion

As a resource for investigation of technical variation, the laboratories involved in the MAQC project have provided measurements with state-of-the-art replication. Such measurements are of particular interest because the variation observed represents a technological challenge, not a circumstance that can be easily changed by closer attention to the requirements of the measurement protocol.

An overview of the MAQC project is provided in Ref. 15. This reference provides a more complete description of the MAQC data including parts of data set not considered in this paper. This reference also contains figures that depict the MAQC data. The title of this reference is problematical in that it implies that reproducibility is a qualitative property that a measurement system does or does not have. In metrological studies, reproducibility is a quantitative property that describes closeness of the agreement between the results of measurements on the same material. In the case of univariate measurements, reproducibility is usually described by a standard deviation. It is not clear what quantitative summary of the technical variation in microarray assays is sufficiently inclusive of all aspects of this technical variation. As demonstrated herein, statistical modeling can portray some aspects of reproducibility for gene expression assays.

On the basis of statistical modeling, this paper characterizes the technical variation in four ways generally corresponding to the figures. First is the linearity of probe responses, which we characterize by comparison with appearances that can be expected from noise alone. Second is the amount of reduction of technical variation by off-the-shelf normalization, which we characterize by comparison with parametric normalization. Third is a fundamental limitation of normalization, which we characterize in terms of sources of variation not subject to the usual normalization assumptions. Fourth is a difference between laboratories, which we characterize by choosing a certain perspective on this difference and comparing it with the difference between materials A and B. Note that the figures except for Figs. 2 and 3 are each based on a different platform. Each characterization could be applied to each platform giving a total of twenty figures, which we do not display because each characterization gives similar results for each platform.

The predominant features of these characterizations and the statistical models that they reflect can be expected to hold true in future applications of the platforms to biological studies. Prediction of properties of the technical variation that one should expect in future studies is the purpose of inter-laboratory studies such as the MAQC project. This standard should be applied to the depiction of the MAQC data in Ref. 15.

Common in the application of microarrays is screening for a few differentially expressed genes. This paradigm influences the way people analyze measurements when trying to understand technical variation. In particular, many of the graphical methods familiar in microarray analyses are centered on quantities with familiar meanings in the context of individual genes and have probe-to-probe independence as the foundation for their interpretation. In contrast, this paper focuses on assay-to-assay associations that involve many probes. Such associations generally point to a few sources of technical variation and, in addition, form the basis for normalization. The characteristics of technical variation we have identified are important in common applications of microarray technology, but the connection requires some thought. Moreover, this thought is different from the quality-control thinking that serves so well for univariate measurements. Univariate quality control has only limited application to a measurement method that gives perhaps 50,000 responses at a time.

Many scientists require a transparent link between the original data and analysis results before they are willing to trust a characterization. Since providing this link with a single figure does not seem possible, we have performed further analyses to verify our characterizations. Regarding Fig. 1, for example, the observation that points for site 2 lie below the *x* = *y* line seems to indicate violation of some assumption about the data besides model lack of fit. We have identified violation of the normality assumption as a possible cause. Regarding Fig. 4, we chose to do factor analysis with two common factors. Inclusion of a third common factor adds detail to the results. More broadly, we note that two different models of microarray intensity form the basis of this paper and that this raises the question of which model is more faithful to the physical processes underlying microarray measurements. Although better models give better results, models do not have to be exact to give useful results. Because we do not use the two models in the same context, they can each be useful without their being in agreement.

The analyses in this paper indicate that technical variation in microarray assays might sometimes be reduced through better approaches to normalization, but that reduction of the technical variation itself through improved measurement protocols and through more consistent adherence to these protocols might finally be needed. Moreover, the analyses provided should be considered in developing experimental designs for microarray studies and QC metrics for monitoring study progress. In particular, our analyses show technical variation problems in comparing expression levels across sites and therefore potentially also over long time periods.

## 5. Methods

The original measurements, the responses of the probes before normalization, are the starting point for our approach. This is necessary because our approach involves insight into technical variation gained through study of normalization. We apply a novel normalization method, parametric normalization, as a way of observing non-linearity in the responses of the probes. In portraying the limitations on normalization, we compare among-laboratory variability with within-laboratory variability. This is a comparison we cannot make with measurements subjected to a type of normalization that reduces within-laboratory variation without similar reduction in the among-laboratory variation.

### 5.1 Probe Selection

We selected probes for each of our four analyses following a similar process for each platform. We began with the 12091 probes in the commonly mapped set chosen by the MAQC organizers for inter-platform comparison. For AG1 and GEH, we eliminated probes from this set based on the qualitative calls or on duplication of an identifier in the original data. For AG1, inclusion is based on the value “0” for control type, feature nonuniformity, and saturation. For GEH, inclusion is based on “L” or “G” as the qualitative call. Moreover, we eliminated any probe that does not meet the inclusion criterion for all sixty assays performed with the platform. We entirely eliminated probes with duplicate identifiers. Thus, we begin our selection with 12091, 12091, 11705, and 9378 probes for ABI, ILM, AG1, and GEH, respectively.

For each platform and each material, we next selected the 3000 probes with highest expression level. Consider material A. First, we transformed the intensities to the base 2 logarithmic scale. This required addition of a starter of 100 for AG1 and a starter of 0.3 for GEH. Next, we centered the logarithmic intensities for each assay at their mean. We then computed the mean over the 15 assays for material A and on the basis of this mean selected the 3000 largest intensities. This gives a set of probes we call the A set. Similarly, we obtained the B set, the C set and the D set.

Our lack-of-fit analysis and normalization comparison are based on combining the A set and the B set with an exclusive OR. In other words, the probes in these analyses are either in the A set but not the B set or in the B set but not in the A set. The number of probes is 1982 for ABI and 1984 for ILM. We applied factor analysis to single materials: the probes in set A, set B, set C and set D for materials A, B, C and D, respectively. We applied discriminant analysis to the material A responses from the probes in set A. We then calculated discriminant coordinates for the material B responses from the probes in set A.

### 5.2 Parametric Normalization

Our algorithm for parametric normalization is a combination of iterative reweighted least squares [16] and crisscross regression [17, 18]. The weighting is based on the suggestion of Rocke and Durbin [19].

For regression under the model discussed in Sec. 2, the weights are inversely proportional to the variance $\sigma_{ig}^{2}$. Following Rocke and Durbin [19], this variance is proportional to the sum of the expression level squared (*x_Ai_θ_Ag_ + x_Bi_θ_Bg_*)^2^ and a constant. We followed the approach of Rocke and Durbin [19] in finding the size of the constant relative to the size of the squared expression level.

To obtain an initial estimate of the weights, we estimate the expression levels from the un-normalized intensities *y_ig_* using un-weighted least squares. Then, using these weights, we re-estimate the expression levels.

The main iteration in our algorithm follows: First, we use the current estimate of the expression levels to estimate the normalization coefficients *η*_0_*_i_* and *η_i_*. These estimates are obtained by fitting the model

$$\eta_{0i}+\eta_{i}(x_{Ai}{\hat{\theta }}_{Ag}+x_{Bi}{\hat{\theta }}_{Bg})$$

to the un-normalized intensities with weighted least squares based in the current expression levels. Second, we re-normalize the intensities

$$\frac{y_{ig}-{\hat{\eta }}_{0i}}{{\hat{\eta }}_{i}}$$

and use these to re-estimate the expression levels. This estimation is based on weights computed from the previous expression levels. We then begin the next iteration by re-estimating the normalization.

### 5.3 Factor Analysis

Separately for each material, we apply factor analysis to original AG1 intensities transformed to the log (base 2) scale. For the 3000 probes with highest expression levels that are given by sets A, B, C, and D, respectively, it is not necessary to add a starter to make the responses all positive. Our factor analysis computations are based on the usual maximum likelihood algorithm [20].

### 5.4 Discriminant Analysis

We apply discriminant analysis to original GEH intensities transformed to the log (base 2) scale with a starter of 0.3 added. We select the set A probes for both materials A and B. Because the starter is necessary for only one intensity value, we could as easily use another approach, but this would not lead to any noticeable change in Fig. 5. The classical approach to discriminant analysis does not apply in our situation because there are many more probes than replicate assays. For this reason, our derivation is based on penalized discriminant analysis [13], which entails adoption of a penalty function. As inputs to their algorithm, we take the centered log base 2 intensities. This centering is an elementary form of normalization. Our choice of penalty function is the identity matrix multiplied by 1000. Hastie and Tibshirani [14] provide a needed part of the algorithm.

## Figures and Tables

**Table 1:** For AG1 results on material A, estimated parameters of the factor analysis model given in Eq. 3. For each assay, the parameters give the distribution of the log (base 2) intensities.

Site 1	Center *µ_i_*	Specific Factor $\psi_{i}^{1/2}$	Common Factor *λ_i_*_1_	Common Factor *λ_i_*_2_
Site 1	9.82	0.26	0.66	0.72
Site 1	9.94	0.25	0.75	0.64
Site 1	9.83	0.25	0.71	0.68
Site 1	9.98	0.23	0.74	0.65
Site 1	9.78	0.25	0.73	0.65
Site 2	10.77	0.07	0.81	0.59
Site 2	10.42	0.07	0.82	0.57
Site 2	10.40	0.09	0.83	0.55
Site 2	10.42	0.16	0.82	0.56
Site 2	10.32	0.09	0.85	0.52
Site 3	10.58	0.10	0.56	0.82
Site 3	10.60	0.11	0.56	0.83
Site 3	10.30	0.04	0.56	0.83
Site 3	10.50	0.08	0.55	0.83
Site 3	10.26	0.07	0.58	0.81

## Footnotes

1 Certain commercial equipment, instruments, or materials are identified in this paper to foster understanding. Such identification does not imply recommendation or endorsement by the National Institute of Standards and Technology, nor does it imply that the materials or equipment identified are necessarily the best available for the purpose.
